# Ferroptosis as a novel therapeutic target for cardiovascular disease

**DOI:** 10.7150/thno.54113

**Published:** 2021-01-01

**Authors:** Xiaoguang Wu, Yi Li, Shuchen Zhang, Xiang Zhou

**Affiliations:** Department of Cardiology, The Second Affiliated Hospital of Soochow University, Suzhou, China

**Keywords:** ferroptosis, cardiomyopathy, myocardial infarction, ischemia/reperfusion injury, heart failure

## Abstract

Cell death is an important component of the pathophysiology of cardiovascular disease. An understanding of how cardiomyocytes die, and why regeneration of cells in the heart is limited, is a critical area of study. Ferroptosis is a form of regulated cell death that is characterized by iron overload, leading to accumulation of lethal levels of lipid hydroperoxides. The metabolism of iron, lipids, amino acids and glutathione tightly controls the initiation and execution of ferroptosis. Emerging evidence shows that ferroptosis is closely associated with the occurrence and progression of various diseases. In recent years, ferroptosis has been found to play critical roles in cardiomyopathy, myocardial infarction, ischemia/reperfusion injury, and heart failure. This article reviews the mechanisms by which ferroptosis is initiated and controlled and discusses ferroptosis as a novel therapeutic target for various cardiovascular diseases.

## Introduction

Over recent years, several forms of regulated cell death have been shown to be involved in the pathogenesis of cardiovascular diseases (CVDs) 1. Caspase-dependent apoptosis was the first mode of regulated cell death to be discovered 2, and in the ensuing decades, accounted for most of the research into cell death. Recently, autophagy has been identified as an evolutionarily conserved lysosomal-dependent pathway for degrading cytoplasmic proteins, macromolecules and organelles, which eventually leads to cell death 3. Ferroptosis is an iron-dependent form of regulated cell death that is characterized by the accumulation of lipid hydroperoxides to lethal levels, resulting in oxidative damage to cell membranes and is recognized to differ from apoptosis, necroptosis and autophagy in several aspects 4-6.

Ferroptosis can be activated by iron overload or by inactivation of glutathione peroxidase 4 (GPX4), the major endogenous mechanism for preventing peroxidation 7-9, which converts potentially toxic lipid hydroperoxides into non-toxic lipid alcohols 10. In the latter case, ferroptosis can be suppressed by activating GPX4. Iron metabolism and activity of GPX4 are thus two major pathways that regulate sensitivity to ferroptosis.

The molecular mechanisms underlying ferroptosis, especially which cell membranes are damaged to cause cell death, remain largely unknown. The morphology of cells that have undergone ferroptosis—which differs from other forms of cell death, such as apoptosis and necrosis—includes dense and compact mitochondria without cristae and loss of plasma membrane integrity. These characteristic morphological features are used as markers of ferroptotic cell death 4. Close links between ferroptosis and pathological processes, including degenerative and neoplastic diseases and ischemic injury, have recently been uncovered 11,12. Ferroptosis has been shown to be involved in drug-induced liver damage 13, acute kidney injury 14,15, neuronal death 16, and cancer cell death 17. Doxorubicin (DOX)-induced ferroptosis in cardiomyocytes causes distortion and enlargement of the myocardial mitochondria 18. Ferrostatins, liproxstatins and many other inhibitors of ferroptosis have been shown to protect the liver, kidney 15, brain 19 and heart 20 in mouse models of ischemic injury. These inhibitors can also reduce symptoms in animal models of degenerative brain disorders including Parkinson's disease 21,22 and Alzheimer's disease 23.

The mechanism of ferroptosis was first described in cells of the central nervous system and shown to be distinct from that of apoptosis. Before introduction of the term 'ferroptosis', this type of cell death was termed 'oxidative glutamate toxicity' or 'oxytosis' 24. Neurological and neoplastic diseases have, for many years, been the focus of both research into the mechanism of ferroptosis and clinical applications. Recent studies have, however, uncovered the links between ferroptosis and CVDs. Ferroptosis is now known to play critical roles in cardiomyopathy, myocardial infarction (MI), ischemia/reperfusion injury (IRI), and heart failure (HF). Suppressing ferroptosis and thus preventing cardiac cell death is likely to become an effective therapeutic strategy for CVDs.

## Mechanisms of ferroptosis

**Figure 1:**
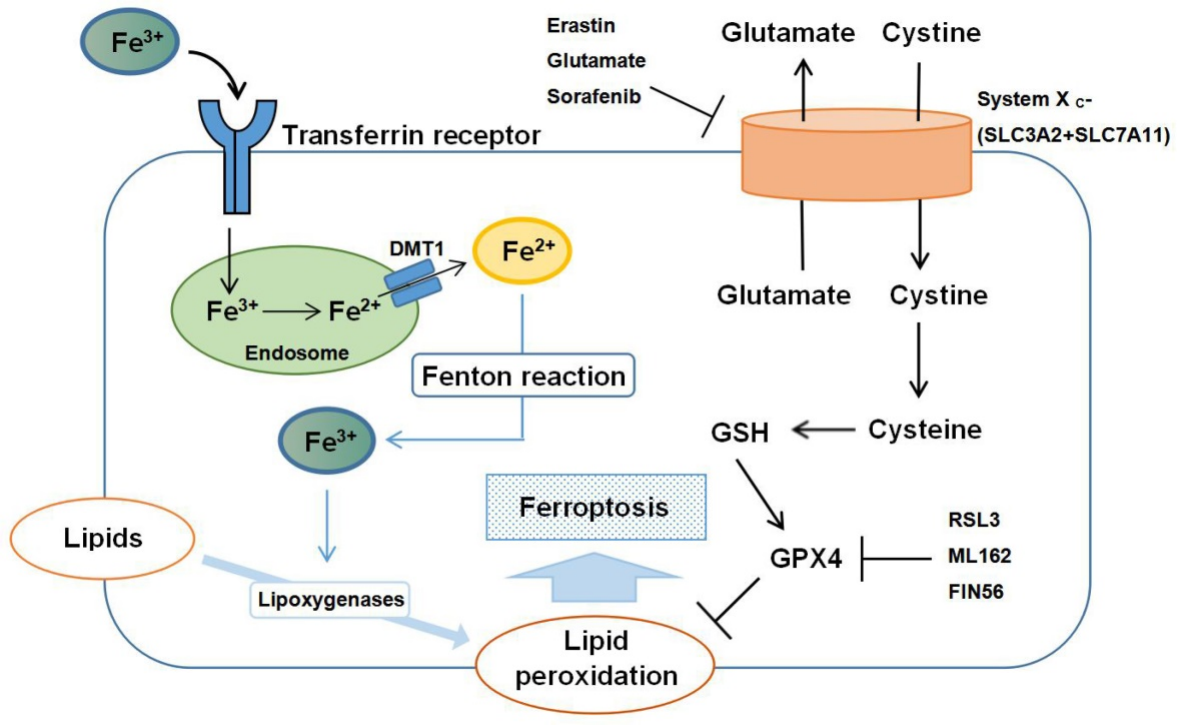
** Schematic representation of the mechanism of ferroptosis.** Ferroptosis is an iron-dependent form of regulated cell death mediated by lipid peroxidation of cellular membranes. Fe^3+^ imported through the transferrin receptor is converted to Fe^2+^ in endosomes and released from endosome by divalent metal transporter 1 (DMT1). Fenton reaction converts Fe^2+^ into Fe^3+^, which induces lipid peroxidation by activating lipoxygenases. Glutathione peroxidase 4 (GPX4) is the major endogenous mechanism to suppress lipid peroxidation. High extracellular concentrations of glutamate inhibit system X_c_^-^, which imports cystine by exchanging intracellular glutamate for extracellular cystine. Cystine is subsequently converted to cysteine, which generates glutathione (GSH), a cofactor for GPX4. Erastin, glutamate, and sorafenib are inhibitors of system X_c_^-^; RSL3, ML162 and FIN56 are inhibitors of GPX4.

The regulatory mechanisms of ferroptosis are complicated, involving a variety of signaling molecules and metabolic pathways **(Figure 1)**. In this review, we summarize the important roles of iron, amino acid, and lipid metabolism in the pathogenesis of ferroptosis.

### Iron metabolism

Iron is imported into the cell from the extracellular space through the transferrin receptor, and transferrin and the transferrin receptor are thus necessary for intracellular accumulation of lipid peroxides and ferroptosis 20. Iron imported into cells by transferrin is in the form of ferric ion (Fe^3+^), which is converted to ferrous ion (Fe^2+^) by ferric reductases in the endosome and transported from the endosome to the cytosol by divalent metal transporter 1. Shuttling of Fe^2+^ to Fe^3+^ via the Fenton reaction contributes to lipid peroxidation and the generation of reactive oxygen species (ROS) 25. Iron-sulfur clusters (Fe-S), which are formed from iron and sulfur, play a critical role in mitochondrial respiration 26, and autophagy has been shown to regulate sensitivity to ferroptosis by an effect on intracellular iron metabolism 27. Yu et al. 28 also demonstrated that the metal transporter Slc39a14 acts as a transporter for non-transferrin-bound iron (NTBI). Slc39a14 is not a major iron transporter under physiological conditions, but can function as a transporter of NTBI in the absence of transferrin. These findings suggest that Slc39a14 may induce ferroptosis by importing iron into the cell when transferrin is depleted.

A number of regulatory proteins and receptors, together with other proteins, can regulate the initiation and execution of ferroptosis by altering lipid peroxidation and mitochondrial function through the control of iron metabolism. For example, heat shock protein beta-1 (HSPB1) and CDGSH iron sulfur domain 1 (CISD1) are both negative regulators of ferroptotic cancer cell death. Knockdown of HSPB1 enhances erastin-induced ferroptosis 29 and genetic inhibition of CISD1 enhances erastin-induced ferroptosis by increasing intra-mitochondrial lipid peroxidation 30. The master regulator, iron responsive element binding protein 2, together with the specific cargo receptor, nuclear receptor coactivator 4, which recognizes ferritin, can regulate sensitivity to ferroptosis by controlling iron metabolism 31. Selective autophagy of ferritin increases sensitivity to ferroptosis by regulating the availability of iron within cells 32.

### Amino acid metabolism

GPX4 plays an indispensable role in the regulation of ferroptosis. Ferroptosis can be initiated both by direct inhibition of GPX4 and by inhibition of synthesis of glutathione (GSH), an essential cofactor for GPX4 function. Extracellular cystine is exchanged for intracellular glutamate in a 1:1 ratio by the plasma membrane cystine/glutamate antiporter, system X_c_^-^, and then converted to cysteine, which is required to generate GSH. Suppression of cysteine uptake leads to accumulation of lipid peroxidation products 33.

High extracellular concentrations of glutamate inhibit system X_c_^-^ and deplete intracellular cystine. Other molecules, such as erastin, sulfasalazine and sorafenib can also deplete intracellular cystine by inhibiting system X_c_^-^. Reduced levels of cystine ultimately inactivate GPX4 by depleting GSH, and initiate ferroptosis 34. Supplementing cells with direct inhibitors of GPX4, such as RSL3, ML162 and FIN56, can initiate ferroptosis by inactivating GPX4. Both amino acid metabolism and glutamine synthesis are linked to the regulation of ferroptosis 35. Glutamine can be converted to glutamate by glutaminases (GLS1 and GLS2), which regulates extracellular glutamate concentrations 36. a-Ketoglutarate, a product of the glutamine-fueled intracellular metabolic pathway, can also replace the requirement of ferroptosis for glutamine. When glutamine and a-Ketoglutarate are depleted, ferroptosis will thus occur because of accumulation of lipid peroxides and ROS, which is caused by depletion of cystine 20.

It is interesting that one of the glutaminases, GLS2, is a transcription target of the tumor suppressor p53, which can suppress ferroptosis by blocking DPP4 activity and limiting DPP4-mediated lipid peroxidation 37. Upregulation of GLS2 thus contributes to p53-dependent ferroptosis 38.

### Lipid metabolism

Polyunsaturated fatty acid (PUFA)-containing phospholipids are the major substrates of ferroptotic lipid peroxidation 39. Accumulation of phosphorlipid hydroperoxides, including phosphatidylcholine, cardiolipin and phosphatidylethanolamine has been detected in ferroptosis. Phospholipids are essential components of biological membranes and the sn-2 position of the phospholipid is always linked to an acyl residue, which is bound to a PUFA. Phospholipids are, therefore, highly susceptible to oxidation because of the PUFA attached to the sn-2 position 40. The addition of PUFA units to the sn-2 position of phospholipids by esterification is catalyzed by polyunsaturated fatty-acid-acyl-CoA (PUFA-CoA).Acyl-CoA synthase long-chain family member 4 (ACSL4), an enzyme involved in phospholipid metabolism, thus affects ferroptosis by catalyzing the formation of PUFA-CoA 41, and inhibition of ACSL4 reduces phospholipid-PUFA. Compared with wild-type cells, in ACSL4 deficient cells, levels of free oxygenated PUFAs are significantly higher than those of esterified oxygenated PUFAs. Inhibition of ACSL4 protects cells from ferroptosis induced by RSL3, a direct inhibitor of GPX4. Notably, ACSL4-deficient cells were not resistant to cell death initiated by other inducers 42.

Another enzyme involved in lipid metabolism, lysophosphatidylcholine acyltransferase 3 (LPCAT3), is closely associated with phospholipid remodeling, and knockdown of LPCAT3 contributes to suppression of ferroptosis 43. It has also been shown that lipoxygenases (LOXs) contribute to ferroptosis by driving the oxidation of arachidonic acid and adrenic acid esterified phosphatidylethanolamines in the endoplasmic reticulum 39. Inhibitors of LOXs, including flavonoids and members of the vitamin E family, can suppress ferroptotic cell death in some contexts 44.

## Ferroptosis and CVDs

In this review, we summarize the association between ferroptosis and diverse CVDs. Iron therapy has been used to relieve clinical symptoms and improve prognosis in patients with HF 45. Excess iron is, however, known to be cardiotoxic. Several recent studies have confirmed that ferroptosis is a potential therapeutic target for various CVDs, including cardiomyopathy, MI, myocardial IRI, and HF** (Table 1)**.

### 3.1 Ferroptosis and cardiomyopathy

**Figure 2:**
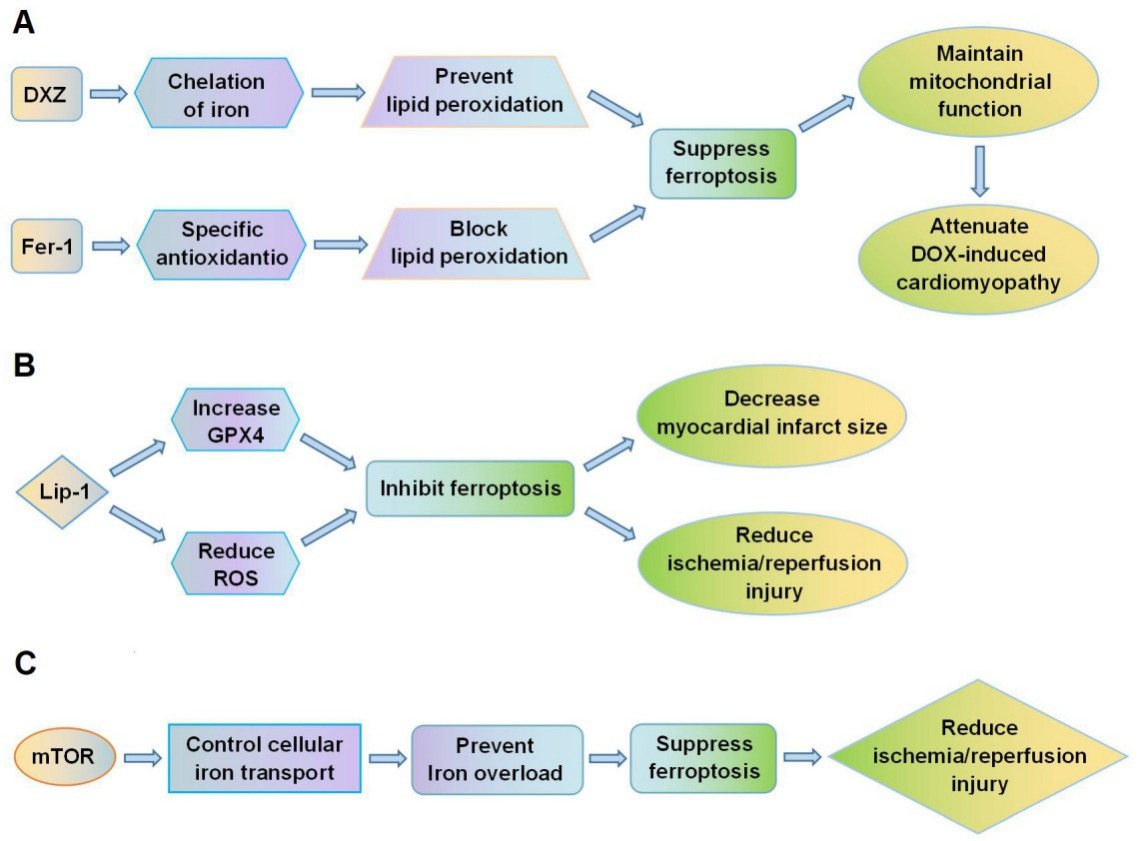
** Ferroptosis is a potential therapeutic target for cardiovascular diseases. (A**) DXZ and Fer-1 protect against DOX-induced cardiomyopathy by suppressing lipid peroxidation, reducing ferroptosis, and maintaining mitochondrial function; (**B**) Lip-1 inhibits ferroptosis by increasing GPX4 expression and decreasing ROS levels, thus reducing myocardial infarct size and ischemia/reperfusion injury. (**C**) mTOR regulates cellular iron transport and prevents iron overload in cardiomyocytes, thus suppressing ferroptosis and reducing ischemia/reperfusion injury. DOX, doxorubicin; DXZ, dexrazoxane; Fer-1, ferrostatin-1; GPX4, glutathione peroxidase 4; Lip-1, liproxstatin-1; mTOR, mechanistic target of rapamycin; ROS, reactive oxygen species.

Fang et al. 18 showed that ferroptosis plays a crucial role in the cardiomyopathy induced by DOX. In mice, administration of DOX led to accumulation of iron in mitochondria and lipid peroxidation of the membranes. Both the ferroptosis inhibitor, ferrostatin-1 (Fer-1), and the iron chelator, dexrazoxane (DXZ), significantly reduced DOX-induced cardiac injury and mortality, whereas inhibitors of apoptosis, necroptosis and autophagy did not improve the condition of the mice. Fer-1 and DXZ afford protection against DOX-induced cardiomyopathy by maintaining the function of mitochondria **(Figure 2A)**. The myocardial mitochondria in mice treated with DOX were distorted and enlarged, and these effects were rescued by both Fer-1 and DXZ. By suppressing ferroptosis, the compounds also reduced infarct size and serum markers of myocardial injury following ischemia/reperfusion. In addition, Fang et al. 18 also found MitoTEMPO, a mitochondrially targeted antioxidant, suppressed ferroptosis by reducing lipid peroxidation, which could eventually attenuate DOX-induced cardiomyopathy. Moreover, RNA-sequencing showed that heme oxygenase-1 (*Hmox1*) was significantly up-regulated in murine heart tissues by DOX-induced ferroptosis. Expression of Hmox1 is upregulated by activation of the redox-sensitive transcription factor, nuclear factor erythroid 2-related factor 2 (Nrf2). DOX augments the Nrf2/Hmox1 pathway, leading to degradation of heme and release of free iron in the heart. Inhibition of ferroptosis could thus provide protection against cardiomyopathy.

### Ferroptosis and MI

Park et al. 46 showed that ferroptotic death of cardiomyocytes occurs during MI. Proteomic analysis of mouse cardiac tissues following MI by left anterior descending ligation showed that protein levels of the ferroptosis inhibitor GPX4 were significantly downregulated in the early and middle stages of MI. Inhibition of GPX4 was shown to sensitize primary neonatal rat ventricular myocytes to ferroptosis when GSH levels were reduced by cysteine deprivation. Nrf2 and epithelial-mesenchymal transition (EMT), which are associated with ferroptosis, were found to be involved in MI. Nrf2 is the master transcription factor that regulates antioxidant responses and suppresses ferroptosis in various types of cells by protecting the cells from lethal ROS stress 47,48. EMT may be linked to sensitivity to ferroptosis by lipogenic reprogramming mediated by ZEB1 49. Hmox1 activity was shown to be enhanced via the Nrf2/Hmox1 pathway in the early and middle stages of MI, leading to the iron excess that contributed to ferroptosis in cardiac cells. EMT signaling is activated during MI to provide a favorable environment for ferroptosis within myocardial cells. Furthermore, Bulluck et al. 50 showed that residual myocardial iron may be a potential therapeutic target to reduce adverse left ventricular remodeling in patients with reperfused MI.

### Ferroptosis and myocardial IRI

Feng et al. 51 showed that liproxstatin-1 (Lip-1) protects against IRI in the mouse myocardium. Lip-1 can reduce the accumulation of ROS generated by lipid peroxidation and strongly inhibit ferroptosis. Post-ischemic administration of Lip-1 was found to reduce myocardial infarct size and protect the integrity of mitochondrial structure. Lip-1 treatment also increased levels of GPX4 protein and reduced levels of ROS. Specifically, Lip-1 protected the mouse myocardium against IRI by suppressing ferroptosis through increasing levels of GPX4 **(Figure 2B)**.

Li et al. 52 indicated that ferroptosis is associated with diabetes myocardial IRI. In diabetic rats with IRI, inhibiting ferroptosis reduced endoplasmic reticulum stress (ERS) and mitigated myocardial damage. The ferroptosis inhibitor Fer-1 also alleviated myocardial damage in H9c2 cells in a high glucose environment, and prevented H9c2 cell damage during hypoxia/reoxygenation. Taken together, these data show that ferroptosis plays a critical role in diabetes myocardial IRI by reducing ERS.

Baba et al. 53 demonstrated that ferroptosis is highly implicated in cardiomyocyte death. Following ischemia/reperfusion in the adult mouse heart, iron accumulated in cardiomyocytes around the myocardial scars. Excessive iron caused cardiomyocyte death and this could be suppressed by inhibiting the production of lipid-derived ROS. The mechanistic target of rapamycin (mTOR) was found to be important in protecting cardiomyocytes from ferroptotic cell death **(Figure 2C)**. mTOR targets multiple iron transport proteins, regulates transferrin receptor 1, and increases the expression of ferroportin 54,55. mTOR could thus affect ferroptosis by controlling iron metabolism in cardiomyocytes.

### Ferroptosis and HF

Chen et al. 56 indicated that cardiac cell death during HF can be reduced by inhibiting autophagy and ferroptosis. Integrated bioinformatical analysis of rat cardiac tissue after HF revealed that TLR4 and NADPH oxidase 4 (NOX4) were two of the up-regulated differently expressed genes. Moreover, knockdown of either TLR4 or NOX4 by intra-myocardial injection of TLR4-siRNA or NOX4-siRNA lentivirus relieved the symptoms of HF by suppressing autophagy and ferroptosis in cardiac cells.

Puerarin has been used clinically to improve HF, but the specific mechanisms remain unclear. Liu et al. 57 found that puerarin blocked either erastin- or isoprenaline-induced ferroptotic death of myocytes and restored cell viability. Puerarin treatment down-regulated the expression of NOX4 and up-regulated GPX4. In rats with HF caused by pressure overload, treatment with puerarin mitigated HF by inhibiting ferroptosis, increasing striated muscle arrangement and reducing mitochondrial atrophy 57.

### Ferroptosis and other CVDs

Bai et al. 58 showed that erastin damages cardiomyocytes by initiating ferroptosis. Erastin rapidly increased levels of ROS in H9c2 cells and this could be inhibited by Fer-1. Erastin-induced ferroptosis could also be inhibited by overexpression of ENPP2, an enzyme that generates the lipid mediator lysophosphatidic acid (LPA). LPA is a bioactive phospholipid that acts as an autocrine/paracrine messenger through activation of G protein-coupled receptors 59. LPA regulates the augmentation of lipoprotein lipase in cardiomyocytes and plays a crucial role in both acute and chronic ischemic cardiac damage 60,61. Overexpression of ENPP2 promoted migration and proliferation of H9c2 cells and decreased erastin-induced generation of ROS. ENPP2 was thus shown to protect cardiomyocytes from the erastin-induced ferroptosis.

Sampilvanjil et al. 62 found that cigarette smoke extract (CSE) caused ferroptotic cell death of rat vascular smooth muscle cells (VSMCs) and that CSE-induced cytotoxicity could be alleviated by inhibitors of ferroptosis. VSMCs were also shown to be more sensitive to the CSE-induced cytotoxicity than other vascular wall cells. CSE led to depletion of GHS and induced lipid peroxidation, which initiated the ferroptotic death of the VSMCs. By fractionating CSE, Noya et al. 63 identified acrolein and methyl vinyl ketone as the major cytotoxic factors responsible for CSE-induced ferroptosis of VSMCs.

Li et al. 64 reported that ferroptosis was involved in sepsis-induced cardiac injury. A septic cardiomyopathy model was established by injection of lipopolysaccharide (LPS) and used to determine the effects of a ferroptosis inhibitor and ferroptosis inducers on LPS-induced myofibroblasts. The ferroptosis inhibitor Fer-1 alleviated LPS-induced cellular damage, whereas the ferroptosis inducers erastin and sorafenib aggravated LPS-induced cellular damage, confirming that ferroptosis is activated in LPS-induced myofibroblasts 64.

## Discussion

Globally, CVDs are the leading cause of mortality, morbidity and disability, and understanding the pathological processes underlying damage to cardiomyocytes is key to developing a cardioprotective strategy. Over recent years, iron overload has been found to play a pathogenic role in cardiotoxicity, both in animal models and in patients with different cardiopathic conditions. Ferroptosis, an iron-dependent form of regulated cell death, has received increasing attention and has been linked to cardiac pathology. Many studies have shown that ferroptosis occurs in cardiomyopathy, MI and the different stages of HF. Inhibitors of ferroptosis are expected to prevent the occurrence of these diseases and promote the syndrome by suppressing ferroptotic death of cardiac cells.

Cardiovascular magnetic resonance imaging studies have shown that iron around the infarct zone is a risk factor for adverse left ventricular remodeling after acute MI. Since ferroptosis and excess iron are significant causes of cardiomyocyte death, inhibiting ferroptosis is a new strategy to reduce cardiac cell death and improve cardiopathic conditions. Inhibitors of ferroptosis, such as Fer-1 and DXZ, would thus be expected to treat cardiomyopathy and MI and to significantly reduce IRI. From a clinical perspective, inhibition of ferroptosis may be a potential therapeutic option for treating and preventing ischemic heart damage, alleviating adverse cardiac remodeling, reducing the risks after reperfusion therapy and aiding prognosis.

Cardiomyocyte death is a fundamental pathological process. Ferroptosis not only occurs in the early stage of cardiomyopathy but also in the early and middle stages of MI. Although levels of inhibitors of ferroptosis, such as GPX4, are known to be reduced and expression of some other genes is known to be up-regulated during cardiovascular disease, the current testing methods are unsuitable for routine clinical diagnosis. The molecular mechanisms underlying ferroptotic cell death, including which cell membranes are damaged, remain largely unknown. One of the key characteristics of ferroptosis is damage to the mitochondrial structure, but it is not easy to routinely observe these changes in mitochondria. When additional mechanisms of ferroptosis are discovered, it may be possible to introduce tests to determine the initiation and execution of ferroptosis, which may be helpful in the early diagnosis of CVD.

Both ferroptosis and necroptosis contribute to ischemic injury. Linkermann et al. 15 demonstrated that simultaneous prevention of ferroptosis and necroptosis provided maximum benefit against ischemic injury. Some modulators, such as p53, affect both apoptosis and ferroptosis but ferroptosis appears to be independent of other cell death pathways. Ferroptosis proceeds in the absence of key effectors of apoptosis, such as BAX, BAK and caspases. It also proceeds in the absence of the key components of necroptosis, such as MLKL, RIPK1 and RIPK3. Early studies have suggested that regulated forms of cell death contribute significantly to the pathogenesis of CVDs, including cardiomyopathy, MI and HF 65,66. Although it remains unclear whether ferroptosis is an independent form of cell death or whether it acts to amplify necrotic cell death, the molecules that mediate ferroptosis may provide new therapeutic targets for CVDs. Fang et al 67 have done an recent study showed that ferritin H, a spherical heteropolymer that comprises 24 heavy and light chain subunits, can store excess cellular iron and plays a significant role in iron metabolism, as discussed earlier. It was further shown that ferroptosis can be triggered in heart-specific Fth knockout mice. Further ongoing research will clarify the mechanisms underlying the initiation and execution of ferroptosis in the heart.

In summary, ferroptosis is critically involved in the pathogenesis of various CVDs, including cardiomyopathy, MI, myocardial IRI, and HF. With ongoing research, suppression of ferroptosis and prevention of cardiac cell death are likely to become an effective therapeutic strategy for CVDs.

## Figures and Tables

**Table 1:** Treatment strategy of ferroptosis in cardiovascular diseases.

Reagents	Key mechanisms	Protective effects	References
Fer-1 DXZ	Prevent lipid peroxidation	Maintain the function of mitochondrial, reduce endoplasmic reticulum stress, and prevent DOX-induced cardiomyopathy.	18
MitoTEMPO	Scavenge lipid peroxidation specifically in the mitochondria	Attenuate DOX-induced cardiomyopathy	18
Lip-1	Increase GPX4 protein levels and reduce ROS generation	Reduce myocardial infarct size and ischemia/reperfusion injury	51
mTOR	Regulate cellular iron transport and reduce ROS production	Protect cardiomyocytes against excess iron and ferroptosis	53
Puerarin	Regulate the expression of NOX4 and GPX4	Restore cell viability and prevent heart failure induced by pressure overload.	57
ENPP2	Reduce ROS generation caused by erastin	Protect cardiomyocytes from erastin-induced ferroptosis	58

DOX, doxorubicin; DXZ, dexrazoxane; Fer-1, ferrostatin-1; GPX4, glutathione peroxidase 4; Lip-1, liproxstatin-1; mTOR, mechanistic target of rapamycin; NOX4, NADPH oxidase 4; ROS, reactive oxygen species.
